# Bilateral inguinal bladder hernias

**DOI:** 10.1002/ccr3.6075

**Published:** 2022-07-18

**Authors:** Naoya Fujita, Yosuke Ono, Yasuhiro Obuchi, Yuji Tanaka

**Affiliations:** ^1^ Department of General Medicine National Defense Medical College Saitama Japan

**Keywords:** benign prostatic hyperplasia, bladder hernia

## Abstract

Inguinal hernia may contain the bladder as one of its contents, while bilateral inguinal bladder herniation is rare. Urinary obstruction and obesity are associated with increased abdominal pressure and are risk factors of bladder herniation. Clinicians should be aware of the bladder hernia in elderly with chronic dysuria and obesity.

## Abstract 2

Clinicians should be aware of the bladder hernia in elderly with chronic dysuria and obesity.

An 80‐year‐old male patient, whose body mass index was 25.9 kg/m^2^, presented with bilateral groin pain despite no tenderness or palpable masses in either inguinal region. He had an 8‐year history of dysuria due to benign prostatic hyperplasia. Computed tomography showed bilateral inguinal hernias appearing as fluid‐filled structures continuous with the bladder (Figure 1). The pain was not relieved by urination. Open surgical hernia repair was therefore performed.

**FIGURE 1:**
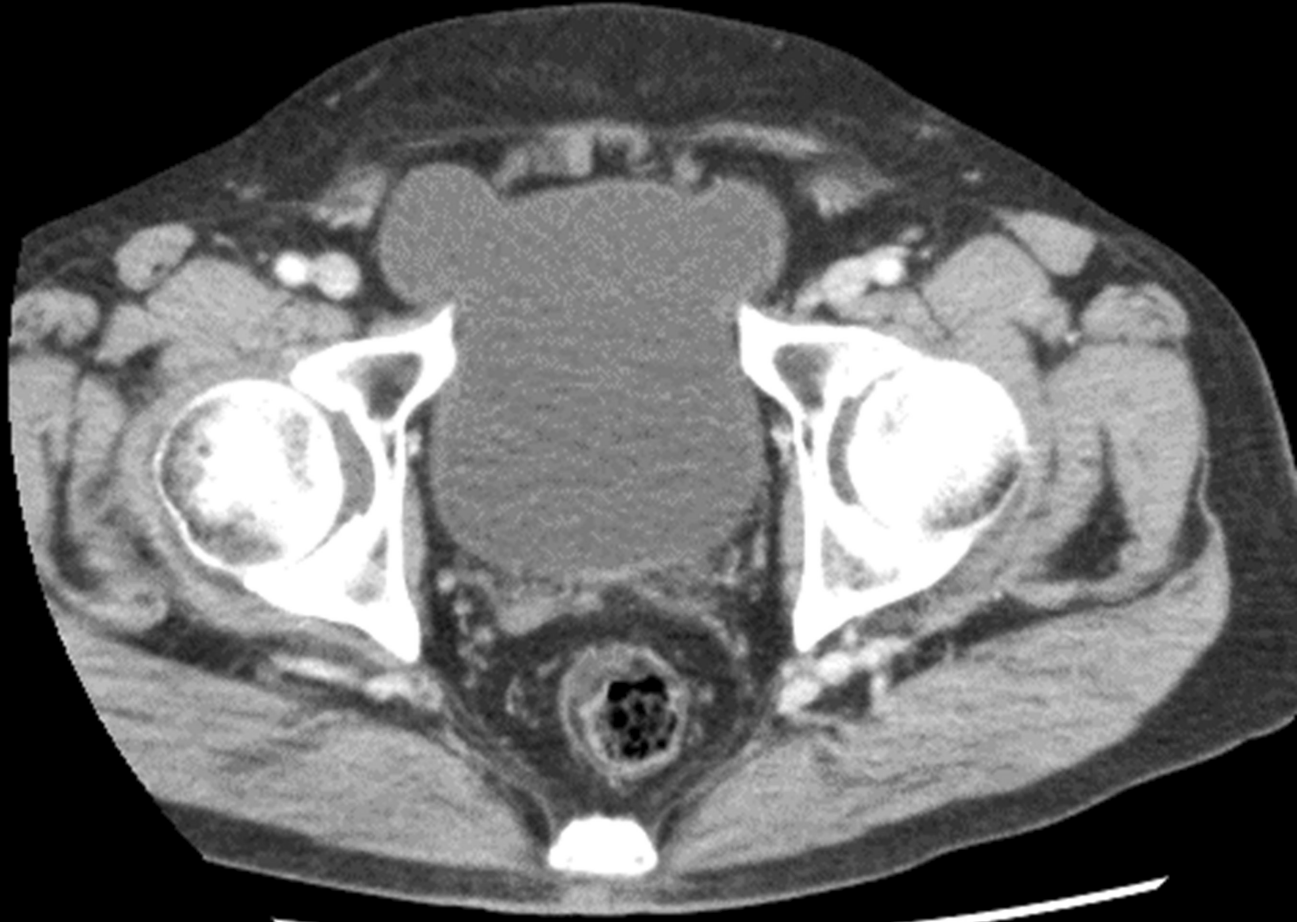
Contrast‐enhanced computed tomography of the abdomen. Bilateral bladder hernias are shown as fluid‐filled structures continuous with the bladder

Inguinal hernia may contain the bladder as one of its contents,^1^ while bilateral inguinal bladder herniation is rare. Horizontal section images of bilateral inguinal bladder hernias are described as “Pelvic Mickey Mouse Sign” because they resemble the Walt Disney character.^2^ Chronic urinary obstruction and obesity are associated with increased abdominal pressure and are risk factors of bladder herniation. Clinicians should be aware of the inguinal bladder hernia as a cause of groin pain in middle‐aged and older male with chronic dysuria and obesity.

## AUTHOR CONTRIBUTIONS

Naoya Fujita wrote the first draft. Yosuke Ono and Yasuhiro Obuchi suggested improvements. Yuji Tanaka revised the manuscript and suggested final changes.

## CONFLICT OF INTEREST

None.

## CONSENT

Written informed consent was obtained from the patient to publish this report in accordance with the journal's patient consent policy.

## Data Availability

None.
